# The Reduction of COMP Serves as a Predictor for Warning of Aortic Dissection Progression

**DOI:** 10.1016/j.jacbts.2025.101329

**Published:** 2025-07-17

**Authors:** Zhuofan Li, Ze Gong, Weihao Li, Yanghui Chen, Rongbo Dai, Shiyu Yang, Yufei Chen, Fang Yu, Yi Fu, Wei Li, Dao Wen Wang, Yiting Jia, Wei Kong

**Affiliations:** a Department of Physiology and Pathophysiology, School of Basic Medical Sciences, Peking University, Beijing, China; b State Key Laboratory of Vascular Homeostasis and Remodeling, Peking University, Beijing, China; c Hwamei College of Life and Health Sciences, Zhejiang Wanli University, Ningbo, China; d Department of Vascular Surgery, Peking University People’s Hospital, Beijing, China; e Division of Cardiology, Department of Internal Medicine and Hubei Key Laboratory of Genetics and Molecular Mechanism of Cardiologic Disorders, Tongji Hospital, Tongji Medical College, Huazhong University of Science and Technology, Wuhan, China; f Department of Pharmacology, School of Basic Medical Sciences, Peking University Health Science Center, Beijing, China

**Keywords:** β-aminopropionitrile, biomarker, cartilage oligomeric matrix protein, Marfan syndrome, thoracic aortic dissection

## Abstract

Thoracic aortic dissection (TAD) is a life-threatening cardiovascular disease that is associated with high morbidity and mortality. The prevailing challenge in TAD monitoring and diagnosis is the lack of novel biomarkers. A significant and sustained reduction in plasma cartilage oligomeric matrix protein (COMP) levels was observed through a case control analysis conducted in 362 TAD patients and 136 controls. The protective role of COMP was assessed in two TAD mouse models. These data suggested that a decreased plasma COMP level might serve as a novel biomarker for TAD progression and COMP could suppress the development of TAD and ameliorate vascular pathogenesis.

## Highlights

• Plasma COMP is a novel biomarker for early detection and prognostic monitoring of TAD.
• The decrease of plasma COMP is a common pathology for nonfamilial TAD and Marfan syndrome.
• Decrease of COMP exacerbated TAD, whereas overexpression of COMP suppressed the development of TAD.

## Visual Abstract

**Figure:**
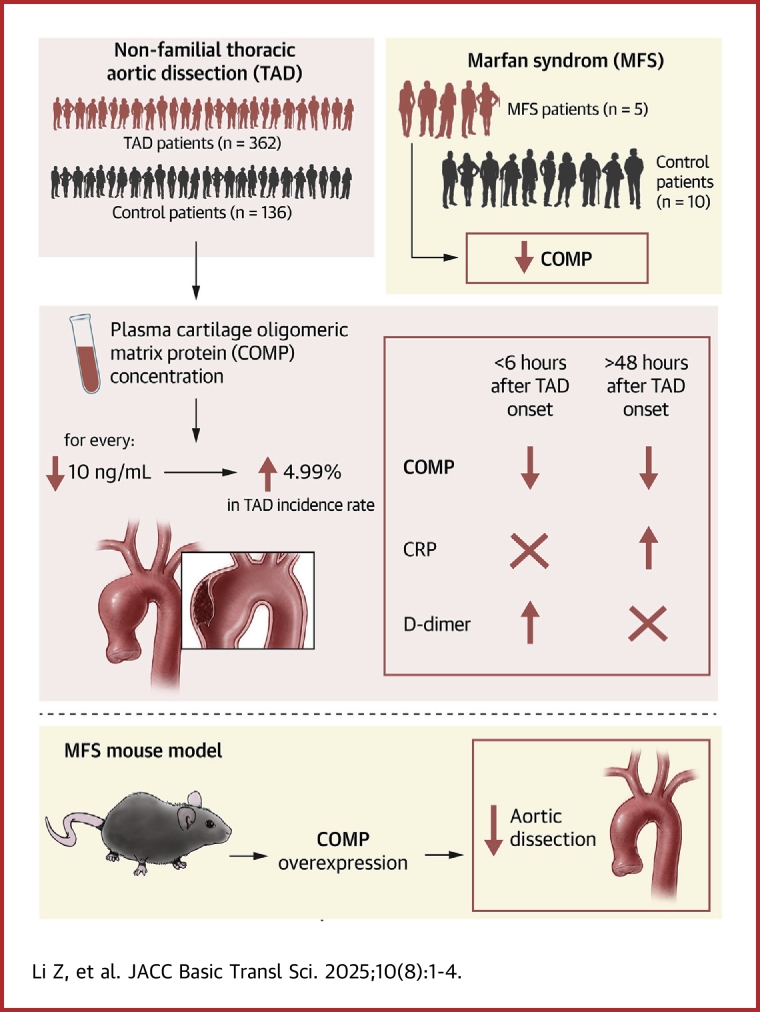

Thoracic aortic dissection (TAD), a highly lethal cardiovascular emergency, has an annual incidence rate of 3.5 to 6.0 cases per 100,000 person-years in the general adult population.^1^^,^^2^ The lack of effective early diagnostic and predictive biomarkers substantially contributes to the high mortality rates observed in clinical TAD management.^3^ TAD is mostly diagnosed using current imaging modalities, including computed tomography and magnetic resonance imaging (MRI) after the occurrence of a false lumen but not during the early stage of TAD.^4^ Thus, a reliable biomarker for early prediction of the pathogenesis and progression of TAD is needed.

Serum biomarkers for TAD have been extensively investigated recently, with C-reactive protein (CRP) and D-dimer emerging as the most-studied candidates.^5^^,^^6^ The established role of CRP in monitoring inflammation suggests that it may have potential diagnostic value for TAD.^7^ However, the increase in CRP levels in infectious and autoimmune conditions or atherosclerotic-related diseases limits its specificity in predicting TAD. Mechanistically, CRP appears to show only a modest association with localized aortic pathophysiological processes, such as elastic fiber degradation or mural inflammation, and may have a limited capacity to discriminate between TAD and other aortic disorders, such as aneurysms, particularly in the early stages of the disease. Additionally, the extended half-life of CRP (18 to 24 hours) may partially limit its utility in the dynamic monitoring of acute aortic events. D-dimer is commonly used as a biomarker to screen for thromboembolic events in conjunction with imaging methods or other biomarkers (eg, matrix metalloproteinase-9 [MMP9]); D-dimer levels alone cannot reliably guide TAD diagnosis, progression monitoring, or risk stratification.^8^^,^^9^ Therefore, rapid, highly specific serological biomarkers that can be used for early diagnosis and prognostic prediction of aortic dissection are urgently needed.

Marfan syndrome (MFS) is a genetic disorder primarily caused by mutations in encoding fibrillin-1 (*Fbn1*), leading to a defective structure and dysfunction of the extracellular matrix (ECM).^10^ Animal models such as *Fbn1*-mutant mice recapitulate key pathophysiological features including aortic wall thinning, elastic fiber fragmentation, and collagen dysregulation.^11^ The key ECM components implicated in MFS pathogenesis include elastin, collagen I/III, and fibrillin-1, and enzymes such as matrix metalloproteinases (MMP2/9) and ADAMTS-7 are associated with ECM degradation.^12^, ^13^, ^14^, ^15^ Despite this knowledge, biomarker development remains challenging: current diagnostic methods rely on *Fbn1* mutation screening, whereas the specificity and utility of potential markers such as desmosine (elastic protein fragments) and hydroxyproline (collagen breakdown product) in monitoring disease progression need to be validated.^16^ Identifying novel and reliable biomarkers is of critical importance for the diagnosis, treatment, and early detection of disease progression in patients with MFS.

In this study, we observed that plasma levels of cartilage oligomeric matrix protein (COMP) exhibited significant sustained decreases from 6 to 48 hours after the onset of TAD, independent of established risk factors such as hypertension and hyperglycemia. This pattern distinguishes COMP from conventional biomarkers such as CRP and D-dimer, suggesting the potential of COMP as a novel biomarker for TAD progression prediction. Furthermore, our investigations in both β-aminopropionitrile (BAPN)–induced aortic dissection and genetically engineered MFS murine models have revealed that COMP exerts robust protective effects against aortic wall pathogenesis. Our findings collectively suggest that COMP could be a potential biomarker for early detection and prognostic monitoring of TAD and may offer innovative strategies for clinical risk stratification and therapeutic intervention.

## Methods

Detailed methodology is described in the Supplemental Material.

### Human study

The human case control study was conducted in accordance with the Declaration of Helsinki and was approved by the Institutional Review Board of Tongji Medical College, Huazhong University of Science and Technology (TJ-IRB20220511). Informed consent was obtained from all participants. A total of 362 TAD patients (including 169 type A TAD patients and 193 type B TAD patients) were consecutively enrolled in this study. The patients were recruited based on the European Society of Cardiology guidelines for the diagnosis and treatment of aortic diseases^17^ and confirmed by computed tomography angiography. All participants successfully completed the inclusion criteria assessment without triggering any exclusion criteria, including aortic trauma, history of congestive heart failure, chronic kidney disease, severe chronic obstructive pulmonary disease, active cancer, and prior aortic surgery. Among those 362 blood samples collected from TAD patients, 72 samples were collected within 6 hours, 132 samples were collected from 6 to 12 hours, 79 samples were collected from 12 to 24 hours, 16 samples were collected from 24 to 48 hours, and 63 samples were collected after 2 days. A total of 136 healthy controls were enrolled from the Health Examination Center, all matched for age and sex with the experimental group.

The study protocol of MFS was approved by Peking University People's Hospital (2022PHB167-001). Informed consent was obtained from all participants. A total of 5 MFS patients were enrolled in this study and diagnosed according to the revised Ghent nosology.^18^ A total of 10 age- and sex-matched healthy controls were enrolled from the Health Examination Center.

A comprehensive dataset comprising demographic variables (sex, age), cardiovascular risk factors (hypertension, smoking history, alcohol consumption patterns), and biochemical markers (fasting plasma glucose) was systematically extracted from electronic health records of the study population. Plasma D-dimer concentrations were quantified using the automated latex-based immunoassay (HemosIL D-dimer HS, Instrumentation Laboratory), whereas plasma CRP levels were determined via latex particle-enhanced immunoassay (CRP Latex Kit, Instrumentation Laboratory). All assays were calibrated against international reference standards to ensure analytical reliability. Blood samples were collected with EDTA as an anticoagulant, followed by centrifugation for 10 minutes at 800 g. Plasma samples were cryopreserved in aliquots of 500 μL using cryovials (Thermo Scientific) at –80 °C (±2 °C deviation) until biochemical analysis.

### Mice

All animal experiments were performed according to the guidelines of the Institutional Animal Care and Use Committee of Peking University Health Science Center. All animals used in this study were raised in the Experimental Animal Science Center of Peking University Health Science Center. C57BL/6J mice were purchased from the Experimental Animal Science Center.

The BAPN-induced model was established with slight modifications. Briefly, 3-week-old mice were fed with 0.3% w/v BAPN in drinking water. The drinking water was refreshed every 2 days. A 28-day BAPN treatment model was used to compare the incidence rate of TAD and related mortality. TAD was defined as thoracic aortic dissection (aortic false lumen formation). In addition, 7- or 14-day BAPN treatment models were used to represent the early stage of TAD formation.

The *COMP*^*−/−*^ mice on a C57BL/6J background were provided by Professor Ake Oldberg (Department of Cell and Molecular Biology, University of Lund, Sweden).^19^ The *Fbn1* ^*C1041G/+*^ mouse line was obtained from George Tellides (Yale University School of Medicine, New Haven, Connecticut, USA)^20^ and established by crossing the *Fbn1*^*C1041G/+*^ male mice with wild-type (WT) C57BL/6J female mice. The *COMP*^*SM-Tg*^ mice were created on a C57BL/6J background by excising the sequence of the TAGLN promoter linked with the mouse COMP cDNA sequence from the vector before pronuclear injection into fertilized C57BL/6J mouse oocytes. And the *Fbn1* ^*C1041G/+*^ *COMP*^*SM-Tg*^ mouse line was generated by crossing *Fbn1*^*C1041G/+*^ male mice with *COMP*^*SM-Tg*^ female mice.

All animals were anesthetized with 1.25% w/v avertin before being sacrificed. For plasma samples, blood was collected from the heart with EDTA as an anticoagulant, followed by centrifugation for 10 minutes at 800 g. For arterial protein and RNA samples, mice were perfused with ice-cold phosphate-buffered saline, and then the thoracic aorta was isolated. For arterial morphology studies, mice were successively perfused with 10% KCl, phosphate-buffered saline, and 4% paraformaldehyde. Then, the whole aorta was isolated, and the ascending aorta (or vascular segment with the most obvious lesion) was used to prepare frozen sections.

### Statistical analysis

All data are shown as the mean ± SEM. Statistical analyses were performed using GraphPad Prism version 8.0 (GraphPad Software) and SPSS version 27.0 (IBM). Normality assessment was performed with Kolmogorov-Smirnov test and the Shapiro-Wilk normality test. For 2-group comparisons of normally distributed data, Student’s *t* test was used. For 2-group comparisons of non-normally distributed data and nonconsecutive data, the Mann-Whitney test was performed. The Pearson correlation test was used to examine the association of 2 continuous variables or a continuous and dichotomous variable (point-biserial correlation). In addition, the Fisher exact test was used for contingency data. One- or two-way analysis of variance (ANOVA) or Kruskal-Wallis test, followed by Sidak’s or Dunn’s post hoc test, respectively, for multiple pairwise comparisons was used for multiple group analyses. In all analyses, a 2-sided *P* < 0.05 was regarded as statistically significant. The diagnostic performance of COMP in TAD, type A TAD and type B TAD was assessed using the receiver operating characteristic (ROC) area under the curve (AUC).^21^ Univariable and multivariable logistic regression analysis was used to evaluate the association of COMP and baseline characteristics with TAD with results presented as the odds ratio with 95% CI.

## Results

### Plasma COMP levels were decreased in TAD patients

**Figure 1:**
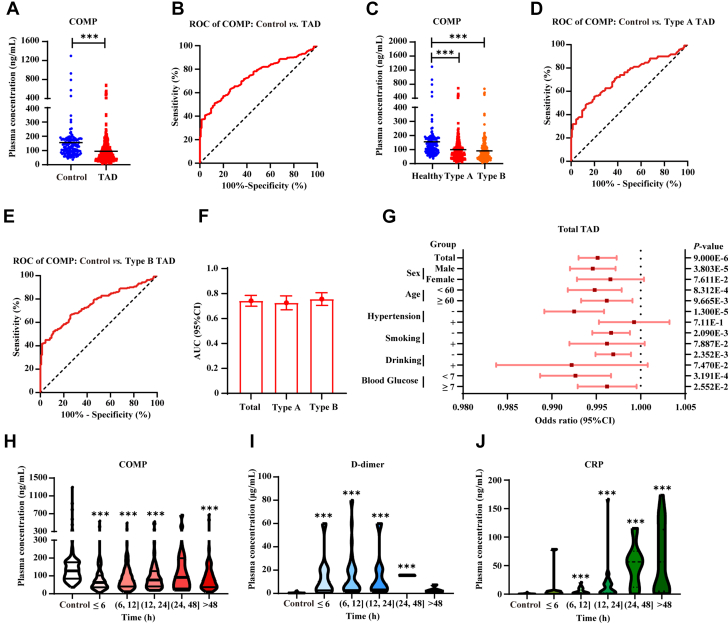
Plasma COMP Levels Were Reduced in TAD Patients (A) Plasma concentration of human cartilage oligomeric matrix protein (COMP) in thoracic aortic dissection (TAD) patients (n = 362) and controls (n = 136). ∗∗∗*P* < 0.001 by Mann-Whitney test. (B) Receiver operating characteristic (ROC) analysis of COMP in total TAD diagnosis. (C) Plasma concentrations of human COMP of type A and type B TAD patients (type A, n = 169; type B, n = 193) and controls (n = 136). ∗∗∗*P* < 0.001 by Kruskal-Wallis test with Dunn’s multiple comparisons test. (D) ROC analysis of COMP in type A TAD diagnosis. (E) ROC analysis of COMP in type B TAD diagnosis. (F) Area under curve (AUC) results of ROC analyses in B, D, and E (show as mean, 95% CI). (G) Odds ratio of human plasma COMP level in TAD diagnosis of different subgroups divided by sex (male, female), age (<60 years, ≥60 years), hypertension (negative, positive), smoking (negative, positive), drinking (negative, positive) and blood glucose (<7 mmol/L, ≥7 mmol/L). (H) COMP levels at different TAD onsets. ∗∗∗*P* < 0.001 by Kruskal-Wallis test with Dunn’s multiple comparisons test compared to control (control, n = 136; time ≤6 hours, n = 72; time >6 hours and ≤12 hours, n = 132; time >12 hours and ≤24 hours, n = 79; time >24 hours and ≤48 hours, n = 16; time >48 hours, n = 63). (I) D-dimer levels at different TAD onsets. ∗*P* < 0.05 by Kruskal-Wallis test with Dunn’s multiple comparisons test compared to control (control, n = 40; time ≤6 hours, n = 19; time >6 hours and ≤12 hours, n = 39; time >12 hours and ≤24 hours, n = 14; time >24 hours and ≤48 hours, n = 2; time >48 hours, n = 6) (J) CRP levels at different TAD onsets. ∗*P* < 0.05 by Kruskal-Wallis test with Dunn’s multiple comparisons test compared to control (control, n = 23; time ≤6 hours, n = 8; time >6 hours and ≤12 hours, n = 17; time >12 hours and ≤24 hours, n = 30; time >24 hours and ≤48 hours, n = 6; time >48 hours, n = 25). All numeric data are shown as the mean ± SEM.

To investigate the potential association between plasma COMP levels and TAD, we conducted a case control study of 362 TAD patients and 136 controls. The clinical demographics and baseline characteristics of the study population are summarized in Table 1. Significant differences were observed between TAD patients and controls, with TAD patients exhibiting increased age (TAD: 53.19 ± 0.56 vs control: 58.05 ± 1.00; *P* < 0.001), systolic blood pressure (*P* < 0.001), and fasting blood glucose levels (TAD: 7.48 ± 0.15 mmol/L vs control: 5.84 ± 0.11 mmol/L; *P* < 0.001). Notably, plasma COMP concentrations were significantly lower in TAD patients than in controls (TAD: 94.47 ± 4.90 ng/mL vs control: 155.50 ± 12.95 ng/mL; *P* < 0.001) (Figure 1A,Table 1). ROC curve analysis revealed that plasma COMP levels were moderately effective at diagnosing TAD, with an AUC of 0.742 (95% CI: 0.698-0.785; *P* < 0.001) (Figure 1B, Supplemental Table 1). These findings show a significant decrease in plasma concentrations of COMP among patients with TAD compared with controls.

**Table 1:** Clinical and Biochemical Characteristics of TAD Patients and Healthy Participants Included in the Study

	TAD (n = 362)	Control (n = 136)	*P* Value
Male	297 (82.0)	102 (75.0)	0.079
Age, y	53.19 ± 0.56	58.05 ± 1.00	<0.001^a^
Hypertension	174 of 362 (48.1)	34 of 99 (25.0)	<0.001^b^
Smoking	106 of 362 (29.3)	29 of 99 (29.2)	1.00
Drinking	58 (16.0)	19 (19.2)	0.45
Blood glucose, n, mmol/L	n = 302; 7.48 ± 0.15	n = 127; 5.84 ± 0.11	<0.001^a^
COMP, ng/mL	94.47 ± 4.90	155.50 ± 12.95	<0.001^a^

Values are n (%) or mean ± SEM.

COMP = cartilage oligomeric matrix protein; TAD = thoracic aortic dissection.

a *P* < 0.05 by Student’s *t* test.

b *P* < 0.05 by chi-square test.

Clinically, aortic dissections were classified according to the tear location: type A aortic dissections originate in the ascending aorta and extend to the aortic root, aortic valve, and coronary artery ostia, whereas type B aortic dissections begin in the descending aorta (distal to the left subclavian artery) and may involve visceral arteries.^17^ Although type A and type B dissections have distinct surgical timing windows, close monitoring of disease progression remains essential for both types of dissection regardless of the initial management strategy used. We investigated whether plasma COMP levels were altered in type A and type B TAD patients by quantifying plasma COMP concentrations in 169 type A TAD patients, 193 type B TAD patients, and 136 controls (Supplemental Table 2). Our analysis revealed significant decreases in plasma COMP levels in both TAD subtypes compared with those in controls. Specifically, type A TAD patients presented a median decrease in COMP levels of 36.4% relative to controls, whereas type B patients presented a 41.7% decrease (type A vs control: 98.84 ± 7.33 ng/ mL vs 155.50 ± 12.95 ng/mL; *P* < 0.001; type B vs control: 90.64 ± 6.59 ng/mL vs 155.50 ± 12.95 ng/mL; *P* < 0.001) (Figure 1C, Supplemental Table 2). Notably, the magnitude of the decrease in COMP levels was not significantly different between the 2 TAD subtypes (*P* = 0.72). ROC analysis revealed an AUC of 0.726 (95% CI: 0.670-0.782; *P* < 0.001) for type A TAD and 0.756 (95% CI: 0.705-0.807; *P* < 0.001) for type B TAD (Figures 1D to 1F, Supplemental Table 1). These results suggested that the plasma levels of COMP were negatively related to both type A and type B TAD.

Pre-existing hypertension, diabetes, smoking, sex (male), age (>50 years), and alcohol consumption are established risk factors for TAD. We subsequently investigated whether these risk factors are correlated with plasma COMP levels. Pearson’s correlation coefficient test indicated that COMP levels were associated with age but not with sex, smoking, alcohol consumption, hypertension, or blood glucose level (Table 2). After adjusting for confounding factors, the plasma COMP level was identified as an independent risk factor for TAD (odds ratio: 0.996; 95% CI: 0.993-0.998; *P* = 0.001) (Table 3). Logistic regression analysis demonstrated that a decreased plasma COMP level was independently associated with a higher risk of TAD in male patients without hypertension, a history of smoking, or alcohol abuse (Figure 1G, Supplemental Table 3). Notably, this association persisted after adjusting for age, sex, and comorbidities, suggesting that a decreased COMP level may be a clinically relevant biomarker for TAD risk stratification.

**Table 2:** Associations of Clinical Variables With COMP

	Correlation Coefficient	*P* Value
Age, y	0.215	<0.001^a^
Sex	–0.024	0.60
Smoking	0.016	0.74
Drinking	0.020	0.66
Hypertension	–0.082	0.068
Blood glucose, mmol/L	–0.027	0.58

Pearson’s correlation coefficient test for age, sex, smoking, drinking, hypertension, and blood glucose.

Abbreviation as in Table 1.

a *P* < 0.05 by Pearson’s correlation coefficient test.

**Table 3:** Logistic Regression Analyses of Circulating COMP Level and TAD

	Unadjusted		Adjusted^a^	
	OR (95% CI)	*P* Value	OR (95% CI)	*P* Value
COMP	0.995 (0.993-0.997)	<0.001^a^	0.996 (0.993-0.998)	0.001^b^

OR = odds ratio; other abbreviations as in Table 1.

a Adjusted for age, hypertension, and blood glucose.

b *P* < 0.05 by logistic regression analysis.

D-dimer and CRP levels are used in current clinical practice to monitor aortic dilation in TAD patients.^5^^,^^7^ Pathologically, increased D-dimer levels reflect active fibrinolysis associated with vascular wall rupture, and CRP accumulation corresponds to the inflammatory response after endothelial injury. However, both biomarkers exhibit limited specificity. D-dimer levels in acute myocardial infarction patients can be 3 to 5 times higher than those in TAD patients, leading to significant false positive rates in TAD prediction.^22^ Moreover, CRP levels are highly susceptible to interference from inflammatory and immune processes, limiting the utility of CRP as a reliable indicator for TAD.^23^ Consequently, no highly specific serological markers for the early detection or risk stratification of TAD have been established. Additionally, to date, no biomarkers for the rapid dilation of the thoracic aorta within a relatively long window of time after acute chest pain occurs in TAD patients have been identified. Therefore, we conducted a longitudinal investigation to examine the long-term changes in plasma levels of COMP, D-dimer, and CRP in patients with TAD after the onset of chest pain symptoms. The results revealed that a decrease in plasma COMP levels occurred within 6 hours of onset, and relatively low levels of COMP were maintained over 48 hours (Figure 1H). Consistent with prior investigations, plasma D-dimer levels rapidly increased in TAD patients, peaking within 24 hours after symptom onset and subsequently decreasing by 48 hours (Figure 1I). In contrast, plasma CRP levels have shown a delayed but sustained increase, reaching their maximum concentration approximately 48 hours after the initial presentation of acute chest pain (Figure 1J). These findings suggest that unlike CRP and D-dimer, COMP may function as a serological biomarker with persistent prognostic value for TAD progression in both the short-term (<6 hours) and long-term (>48 hours) phases after the occurrence of chest pain.

Taken together, these results show that plasma COMP levels were significantly decreased in both type A and type B TAD patients and remained persistently low from 6 hours to more than 48 hours after symptom onset, suggesting that COMP is a novel potential biomarker for TAD diagnosis and prediction of aortic dilatation, exhibiting a distinct temporal profile compared with that of conventional biomarkers.

### COMP was decreased in the aortas of BAPN-induced TAD mice

**Figure 2:**
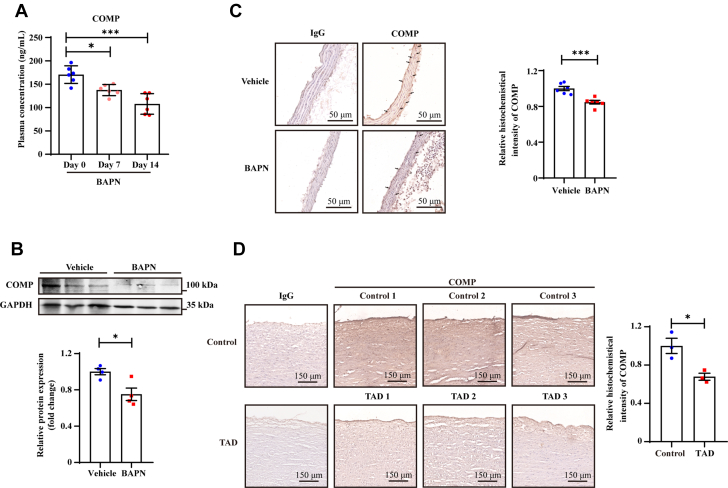
COMP Was Downregulated in BAPN-Induced Mouse TAD and TAD Patients (A) Plasma concentrations of mouse COMP from control (day 0, n = 6) and 7- or 14-day 0.3% β-aminopropionitrile (BAPN)–treated (day 7, n = 6; day 14, n = 6) C57 male mice. ∗*P* < 0.05, ∗∗∗*P* < 0.001 by one-way analysis of variance with Dunn’s multiple comparisons test. (B) Representative western blotting and quantitative analysis of COMP expression in thoracic aortas from 7-day 0.3% BAPN-treated (BAPN) or control (Vehicle) C57 male mice. ∗*P* < 0.05 by Student’s *t* test (n = 4). (C) Representative immunohistochemical staining and quantitative analysis of COMP in control (Vehicle, n = 6) and BAPN-induced TAD samples (BAPN, n = 6). ∗∗∗*P* < 0.001 by unpaired Student’s *t* test (scale bar = 100 μm as shown in the image). (D) Representative immunohistochemical staining and quantitative analysis of COMP in healthy control (control, n = 3) and TAD samples (TAD, n = 3). ∗*P* < 0.05 by Student’s *t* test (scale bar = 100 μm). All numeric data are shown as the mean ± SEM. GAPDH = glyceraldehyde-3-phosphate dehydrogenase; IgG = immunoglobulin G; other abbreviations as in Figure 1.

Our previous studies have identified COMP as a protective vascular ECM protein that maintains vascular homeostasis and counteracts the development of restenosis, atherosclerosis, and aneurysms.^24^, ^25^, ^26^ Given the dramatic reduction in plasma COMP levels observed in TAD patients after symptom onset, we investigated whether COMP expression was altered in the aortic wall during TAD progression. A modified TAD mouse model was generated by administering 0.3% BAPN in drinking water to 3-week-old C57BL/6 mice. Consistent with the findings of the above human study, plasma COMP levels were markedly reduced after 7 or 14 days of BAPN intake (Figure 2A) before the aorta exhibited obvious dissection. COMP levels in the thoracic aorta were significantly decreased after 7 days of BAPN intake, as assessed by western blot analysis and immunohistochemical staining (Figures 2B and 2C). In addition, compared with those of healthy people, decreased COMP was also detected in the aortas of patients with TAD (Figure 2D, Supplemental Table 4).

These findings reveal that the local expression of COMP in the vasculature markedly decreased during TAD progression, with particularly pronounced reductions observed in the early stage of the disease.

### COMP deficiency exacerbated BAPN-induced TAD

To establish a causal relationship between COMP deficiency and TAD pathogenesis, we conducted loss-of-function studies using COMP-knockout (*COMP*^*−/−*^) mice.^19^ Three-week-old male *COMP*^*−/−*^ mice and their WT littermates were used in the BAPN-induced TAD model.

**Figure 3:**
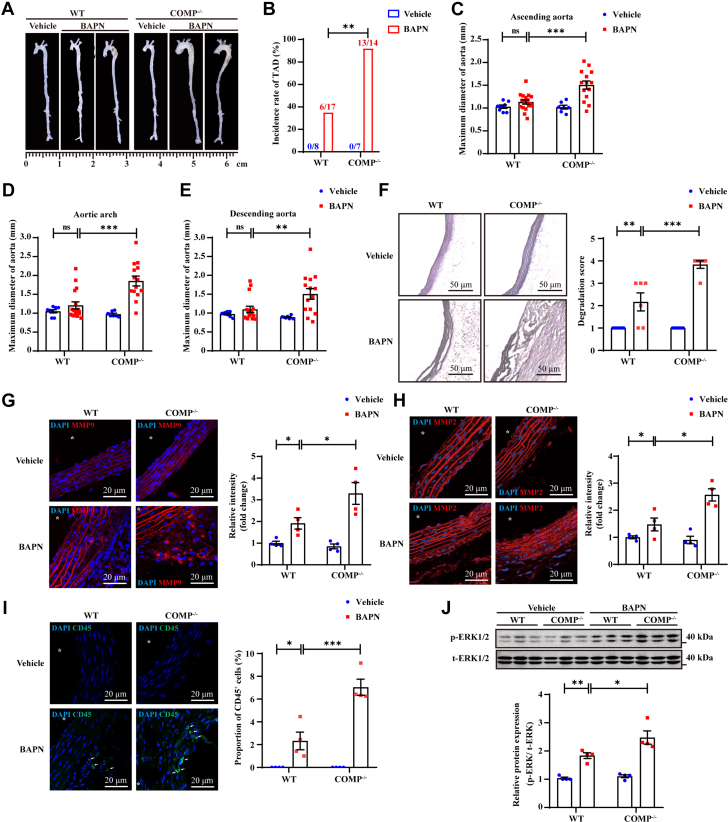
COMP Deficiency Exacerbated BAPN-Induced TAD Two groups of 3-week-old male mice (BAPN: wild-type [WT], n = 17; *COMP*^*−/−*^, n = 14) were treated with 0.3% BAPN in drinking water for 28 days. And another 2 groups of 3-week-old male mice (Vehicle: WT, n = 8; *COMP*^*−/−*^, n = 7) were fed with normal water as non-TAD control. (A) Representative ex vivo images of aorta from 4 groups. Scale bar was shown in the image. (B) Incidence rate of TAD in BAPN-treated mice. ∗∗*P* < 0.01 by Fisher’s exact test. (C to E) Maximum diameter of aorta from male mice was measured ex vivo at 3 segments, including ascending aorta (Asc) (C), aortic arch (Arch) (D), and descending aorta (Des) (E). ∗∗*P* < 0.01, ∗∗∗*P* < 0.001 by 2-way analysis of variance (ANOVA) with Sidak’s multiple comparisons test. (F) Representative images of Elastin van Gieson staining and elastin degradation score. ∗∗*P* < 0.01, ∗∗∗*P* < 0.001 by Kruskal-Wallis test with Dunn’s multiple comparisons test (scale bar = 50 μm as shown in the image). (G to I) Representative immunofluorescence staining and quantitative analysis of matrix metalloproteinase-9 (MMP9) (G), MMP2 (H), and CD45^+^ leukocytes (I) in thoracic aorta from 28-day 0.3% BAPN-treated (BAPN) or control (Vehicle) WT or *COMP*^*−/−*^ male mice. ∗*P* < 0.05, ∗∗∗*P* < 0.001 by Kruskal-Wallis test with Dunn’s multiple comparisons test (scale bar = 20 μm as shown in the image). ∗Indicates lumina. (J) Representative western blotting and quantitative analysis of p-ERK1/2, t-ERK1/2 in thoracic aorta from 7-day 0.3% BAPN-treated (BAPN) or control (Vehicle) WT or *COMP*^*−/−*^ male mice. ∗*P* < 0.05, ∗∗*P* < 0.01 by 2-way ANOVA with Sidak’s multiple comparisons test. All numeric data are shown as the mean ± SEM. ERK = extracellular signal-regulated kinase; other abbreviations as in Figures 1 and 2.

Two of 17 (12%) WT mice died from TAD-related aortic rupture, and COMP deficiency resulted in comparable mortality (n = 2 of 14, 14%) on day 28 after BAPN administration (Supplemental Figures 1A and 1B). Compared with their WT littermates, *COMP*^*−/−*^ mice presented increased TAD formation (WT-BAPN, n = 6 of 17, 35% vs *COMP*^*−/−*^-BAPN, n = 13 of 14, 93%: *P* = 0.002) (Figures 3A and 3B, Supplemental Table 5). In addition, COMP deficiency resulted in marked aortic dilation (ascending aorta, WT-BAPN 1.13 ± 0.05 mm vs *COMP*^*−/−*^-BAPN 1.51 ± 0.09 mm; *P* < 0.001; aortic arch, WT-BAPN 1.21 ± 0.09 mm vs *COMP*^*−/−*^-BAPN 1.85 ± 0.13 mm; *P* < 0.001; descending aorta, WT-BAPN 1.10 ± 0.08 mm vs *COMP*^*−/−*^-BAPN 1.51 ± 0.14 mm; *P* = 0.007) (Figures 3C to 3E). These results suggest that COMP deficiency exacerbates BAPN-induced vascular dilation.

The infiltration of inflammatory cells such as macrophages into the vessel wall and the degradation and disruption of elastic fibers are major pathological hallmarks of TAD pathogenesis.^27^^,^^28^ Elastica van Gieson staining revealed that COMP deficiency significantly promoted elastin degradation in response to BAPN stimulation (WT-BAPN 2.17 ± 0.40 vs *COMP*^*−/−*^-BAPN 3.83 ± 0.17; *P* < 0.001) (Figure 3F). The expression of MMPs, particularly MMP2 and MMP9, combined with inflammatory cell infiltration, are also characteristics of aortic dissection pathogenesis.^29^ The levels of MMP9 as well as MMP2 and recruitment of CD45^+^ leukocytes were markedly increased in the vascular walls of *COMP*^*−/−*^ mice after 28 days of BAPN intake (Figures 3G to 3I). Previous reports have indicated dramatically activated extracellular signal-regulated kinase (ERK) signaling in the early stage of TAD.^30^^,^^31^ Consistently, we observed enhanced ERK1/2 phosphorylation in the aortas of WT mice with BAPN intake (WT-BAPN) compared with those from WT mice with vehicle intake (WT-vehicle) on day 7, which was significantly greater than that in *COMP*^*−/−*^ mice with BAPN intake (*COMP*^*−/−*^-BAPN) (Figure 3J).

COMP deficiency exacerbated BAPN-induced TAD formation.

### Decreased COMP levels were associated with the progression of MFS

MFS is an autosomal dominant connective tissue disease. Genetically, MFS is caused by mutations in the *FBN1* gene, leading to fibrillin-1 deficiency, which induces abnormalities in elastic fiber structure and weakened vascular wall integrity, substantially increasing the risk of aortic dissection.^10^ Approximately 60% to 80% of patients with MFS develop aortic root dilatation, and 20% to 30% of these patients ultimately progress to TAD.^32^ Given the negative relationship between COMP levels and TAD progression, we next assessed whether COMP protects against MFS.

**Figure 4:**
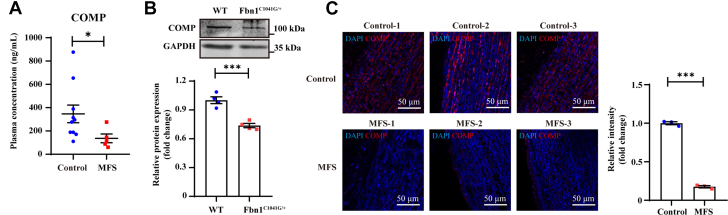
Decreased COMP Levels Were Associated With the Progression of Marfan Syndrome (A) Plasma concentration of human COMP in Marfan syndrome (MFS) patients (n = 5) and controls (n = 10). ∗*P* < 0.05 by Mann-Whitney test. (B) Representative western blotting and quantitative analysis of COMP expression in thoracic aortas from 30-week-old C57 and *Fbn1*^*C1041G/+*^ male mice. ∗∗∗*P* < 0.001 by Student’s *t* test (n = 4). (C) Representative immunofluorescence staining and quantitative analysis of COMP in ascending aortas from healthy controls and MFS patients. ∗∗∗*P* < 0.001 by Student’s *t* test (scale bar = 50 μm as shown in the image). All numeric data are shown as the mean ± SEM. Abbreviations as in Figure 1, Figure 2, Figure 3.

As shown in (Figure 4A, Table 4), plasma COMP levels were significantly lower in MFS patients than in age- and sex-matched controls (MFS: 136.40 ± 37.61 vs control: 346.30 ± 76.14; *P* = 0.021), suggesting a dramatic reduction in COMP levels during the pathogenic progression of MFS. Furthermore, heterozygous *Fbn1*^*C1041G/+*^ mice were used as an MFS mouse model as previously reported.^33^ Consistently, *Fbn1*^*C1041G/+*^ mice presented body weights and blood pressures comparable to those of their WT littermates at the age of 30 weeks (Supplemental Table 6). However, the local expression of COMP within vessel walls was significantly reduced in *Fbn1*^*C1041G/+*^ mice compared with that in WT mice (Figure 4B). Similar to the *Fbn1*^*C1041G/+*^ mice, COMP levels were dramatically decreased in aortas from patients with MFS compared with those from controls (Figure 4C, Supplemental Figure 2A, Supplemental Table 7).

**Table 4:** Clinical and Biochemical Characteristics of MFS Patients and Healthy Participants Included in the Study

	MFS (n = 5)	Control (n = 10)	*P* Value
Male	4 (80.0)	8 (80.0)	>0.99
Age, y	35.20 ± 2.38	35.50 ± 1.41	0.91
Hypertension	3 (60.0)	7 (70.0)	>0.99
COMP, ng/mL	136.40 ± 37.61	346.30 ± 76.14	0.021^a^
ARB/ACEI	3 (60.0)	5 (50.0)	>0.99
β-blocker	2 (40.0)	5 (50.0)	>0.99

Values are n (%) or mean ± SEM.

ACEI = angiotensin-converting enzyme inhibitor; ARB = angiotensin receptor blocker; MFS = Marfan syndrome; other abbreviation as in Table 1.

a *P* < 0.05 by Mann-Whitney test.

Taken together, these findings indicate that a reduction in COMP level is closely associated with the pathogenesis of MFS.

### COMP suppressed the development of aortic dissection in an MFS mouse model

**Figure 5:**
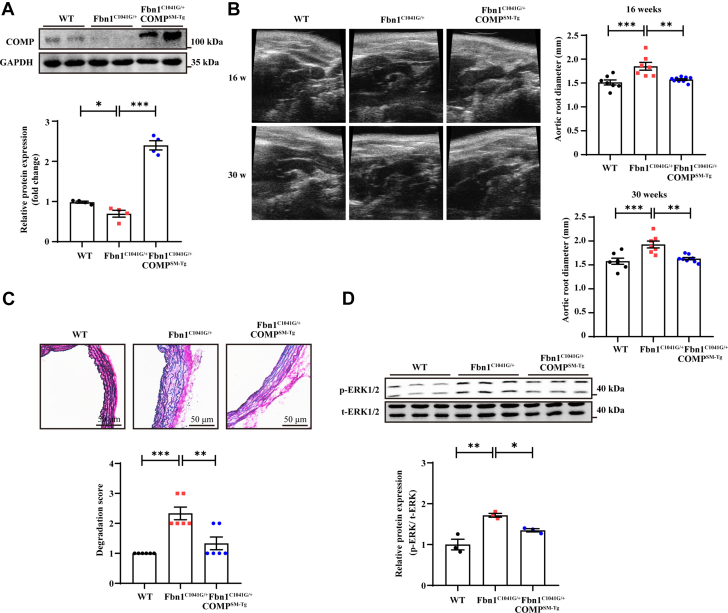
COMP Suppressed the Development of Aortic Dissection in Marfan Syndrome Mouse Model (A) Representative western blotting and quantitative analysis of COMP expression in thoracic aortas from 30-week-old C57, *Fbn1*^*C1041G/+*^, and *Fbn1*^*C1041G/+*^*COMP*^*SM-Tg*^ male mice. ∗*P* < 0.05, ∗∗∗*P* < 0.001 by Student’s *t* test (n = 4). (B) Representative transthoracic echocardiographic images and quantification of diameters of the aortic roots in C57 (n = 6), *Fbn*^*C1041G/+*^ (n = 6), and *Fbn1*^*C1041G/+*^*COMP*^*SM-Tg*^ (n = 9) male mice at 16 weeks and 30 weeks. ∗∗*P* < 0.01, ∗∗∗*P* < 0.001 by 1-way ANOVA with Dunn’s multiple comparisons test. (C) Representative images of Elastin van Gieson staining and elastin degradation score. ∗∗*P* < 0.01, ∗∗∗*P* < 0.001 by Kruskal-Wallis test with Dunn’s multiple comparisons test (scale bar = 50 μm as shown in the image). (D) Representative western blotting and quantitative analysis of p-ERK1/2, t-ERK1/2 in thoracic aorta from 30-week-old WT, *Fbn1*^*C1041G/+*^, and *Fbn1*^*C1041G/+*^*COMP*^*SM-Tg*^ male mice. ∗*P* < 0.05, ∗∗*P* < 0.01 by 1-way ANOVA with Sidak’s multiple comparisons test. All numeric data are shown as the mean ± SEM. Abbreviations as in Figure 1, Figure 2, Figure 3.

To explore the role of COMP in MFS-related TAD pathogenesis, we crossed *Fbn1*^*C1041G/+*^ mice with *COMP*^*SM-TG*^ mice to induce smooth muscle cell–derived COMP overexpression in vivo (Supplemental Figures 3A and 3B). Western blotting analysis revealed significant overexpression of COMP in the aortas of 30-week-old male *Fbn1*^*C1041G/+*^ *COMP*^*SM-TG*^ mice compared with those of their *Fbn1*^*C1041G/+*^ littermates (Figure 5A). Body weight and blood pressure did not significantly differ between *Fbn1*^*C1041G/+*^ *COMP*^*SM-TG*^ mice and *Fbn1*^*C1041G/+*^ mice at the age of 30 weeks (Supplemental Table 8). Transgenic COMP expression reversed aortic dilation in MFS mice at week 30, as assessed by transthoracic echocardiography (Figure 5B, Supplemental Table 8). Accordingly, the elastic lamina breakage in the aortas of Marfan mice was completely reversed by COMP overexpression (Figure 5C). In addition, the phosphorylation of ERK1/2 was significantly enhanced in the thoracic aortas of *Fbn1*^*C1041G/+*^ mice but was reduced in those of *Fbn1*^*C1041G/+*^ *COMP*^*SM-TG*^ mice (Figure 5D). Taken together, these data collectively indicate that COMP suppresses the pathogenesis of TAD in MFS mice.

Our study identified plasma COMP levels as a potential predictive biomarker for the occurrence of both TAD and aortic dilation and demonstrated the protective role of COMP in TAD animal models.

## Discussion

TAD is a challenging disease with limited methods for early diagnosis and inadequate treatment strategies. In this study, we identified a decrease in plasma COMP levels as a novel biomarker for TAD progression. The decreased COMP levels in plasma and the aorta were confirmed by using 2 TAD mouse models. Moreover, TAD formation and development were promoted by COMP deficiency and attenuated in *COMP*^*SM-TG*^ mice. Our study suggests COMP as a novel target for TAD prediction and protection.

The most significant finding of this study is the identification of COMP as a novel indicator for TAD. Plasma COMP levels were consistently reduced in both type A and type B nonfamilial TAD patients. Early-stage TAD cannot be detected by conventional imaging modalities (computed tomography angiography or MRI) before the aorta forms a false lumen or significant dilation occurs, underscoring the urgent need for blood-based biomarkers for early diagnosis.^4^ Although existing markers such as CRP and D-dimer have been studied in aortic diseases, there are critical limitations: CRP and D-dimer lack specificity for TAD and do not capture early pathogenesis, with D-dimer levels rapidly declining within 2 days of symptom onset and CRP levels remaining unchanged during the initial phase.^5^^,^^7^ Some studies have identified novel biomarkers to aid in TAD diagnosis, but large samples to validate these findings are still lacking.^34^^,^^35^ Conversely, our data show a distinct temporal profile of COMP reduction, starting as early as 6 hours after dissection and persisting for ≥48 hours. This early and prolonged decrease in COMP levels distinguishes COMP from conventional biomarkers, suggesting its superior reliability for TAD detection and monitoring.

To mitigate TAD-related mortality in high-risk individuals (eg, those with hypertension or genetic predisposition), early risk prediction using circulating biomarkers is critical. Our logistic regression analysis revealed a significant dose-response relationship: every 10-ng/mL decrease in plasma COMP levels was associated with a 4.99% increase in TAD incidence (Table 3). These findings suggest that COMP could be used as a quantitative risk indicator for TAD development in cardiovascular high-risk populations.

Also known as thrombospondin-5, COMP is abundantly expressed in the vasculature.^36^ Our previous studies have shown that COMP maintains vascular smooth muscle cells and endothelial cell homeostasis, thereby preventing post-injury neointima formation, hypertension, vascular calcification, and atherosclerosis.^24^, ^25^, ^26^^,^^37^^,^^38^ However, the role of COMP in the progression of TAD is still unknown. In this study, we found that *COMP*^*−/−*^ mice presented exacerbated aortic wall remodeling in a BAPN-induced TAD model. In contrast, COMP overexpression alleviated vascular dilation and aortic rupture-associated mortality in a genetically engineered MFS mouse model. These findings collectively suggest that COMP exerts a protective effect on the progression of TAD. These insights are consistent with our previous discovery of the protective role of COMP in vascular integrity and provide a mechanistic basis for translating COMP levels into clinical practice.

TAD arises from a complex interplay of molecular, genetic, and hemodynamic factors that disrupt aortic wall integrity. Degradation of the ECM — primarily driven by enzymes such as matrix metalloproteinases (MMP2/9) which break down collagen and elastin — weakens the medial layer, creating structural vulnerabilities. Simultaneous, chronic inflammation mediated by activated macrophages results in the release of proinflammatory cytokines (eg, interleukin 6 and tumor necrosis factor- α), promoting oxidative stress and fibrotic remodeling.^12^^,^^13^^,^^39^ Genetic predispositions, such as mutations in FBN1 (fibrillin-1) in MSA or COL1A1 (collagen synthesis defects), further compromise ECM stability. Hemodynamic stress due to hypertension induces mechanical fatigue, accelerating medial wall degeneration and loss of elastin elasticity and aberrant collagen deposition impair the ability of the aorta to withstand pulsatile forces, predisposing it to intimal tears.^10^^,^^11^ Although previous reports have revealed that COMP may protect against vascular remodeling by inhibiting the AT1R-mediated β-arrestin-2 pathway, interacting with integrin α_7_β_1_ et cetera,^24^^,^^25^ the precise mechanism by which COMP alleviates the progression of TAD remains incompletely understood and warrants further investigation.

### Study limitations

Although the decrease in plasma COMP levels occurred earlier and persisted longer in TAD patients than that of CRP and D-dimer levels, the ROC curve analysis revealed that COMP is still limited as an independent diagnostic marker. Whether COMP can discriminate patients with TAD from other acute chest pain diseases, such as acute myocardial infarction or pulmonary embolism must be validated in a large sample of patients.

## Conclusions

Our findings provide the first evidence that a decrease in COMP is a novel biomarker of TAD and that COMP could be a potential therapeutic target to prevent TAD progression.

Perspectives **COMPETENCY IN MEDICAL KNOWLEDGE:** TAD is a life-threatening cardiovascular disease associated with high morbidity and mortality. Novel biomarkers for early diagnosis and monitoring of TAD development are still lacking. This study explored the decrease in plasma COMP levels in the early stage of TAD and its maintenance in the long term. The decrease in COMP was associated with the development of TAD in both the BAPN-induced TAD mouse model and the MFS mouse model.

**TRANSLATIONAL OUTLOOK:** Our research suggests that plasma COMP level is a novel biomarker that could aid in the diagnosis of TAD in the early stage before the false lumen occurs and in monitoring the development of TAD in high-risk populations. Furthermore, targeting COMP might be a potential therapeutic strategy for TAD.

## Funding Support and Author Disclosures

This work was supported by grants from the Noncommunicable Chronic Diseases-National Science and Technology Major Project (2024ZD0526400), the National Natural Science Foundation of China (NSFC 82370451), and the Beijing Natural Science Foundation (7224347). The authors have reported that they have no relationships relevant to the contents of this paper to disclose.
