# Supplementary material for: The Reduction of COMP Serves as a Predictor for Warning of Aortic Dissection Progression
Source: JACC Basic Transl Sci. 2025 Jul 17;10(8):101329. doi: 10.1016/j.jacbts.2025.101329 (PMC12296508; doi:10.1016/j.jacbts.2025.101329)
Supplement: Supplemental Material [file mmc1.docx]

**Supplemental material**

**Materials and Methods**

**Materials**

β-aminopropionitrile (BAPN) was purchased from Sigma-Aldrich (A3134, St. Louis, MO, USA) to establish mouse thoracic aortic dissection (TAD) model. Antibody against cartilage oligomeric matrix protein (COMP, ab42225) used for western blotting and immunostaining was purchased from Abcam (Cambridge, UK). Antibody against GAPDH (60004-1-Ig) used for western blotting was obtained from Proteintech (Wuhan, China). Antibody against MMP2 (10373-2-AP) and MMP9 (10375-2-AP) used for immunofluorescence staining was purchased from Proteintech (Wuhan, China). Antibodies against ERK1/2 (9102S) and phospho-ERK1/2^T202/Y204^ (9101S) were obtained from Cell Signaling Technology (Boston, USA). Fluorescein isothiocyanate (FITC) conjugated rat anti-mouse CD45 antibody (103107) used for immunofluorescence staining was purchased from Biolegend (San Diego, USA). Normal rabbit IgG (2729) used as a negative control for immunostaining was purchased from Cell Signal Technology (Boston, USA). Immunohistochemical staining kit (PV-6001) and DAB kit (ZLI-9018) were purchased from ZSGB-BIO (Beijing, China). 4,6-diamidino-2-phenylindole (DAPI) (62248) and Alexa Fluor^TM^ 633 goat anti-rabbit (H+L) antibody (A21070) used for immunofluorescence staining were purchased from Thermo Fisher Scientific (Rochester, USA). Human COMP ELISA kit (DCMP0) was purchased from R&D (Minneapolis, USA). Mouse COMP ELISA kit (SEB197Mu) was purchased from Cloud-Clone (Wuhan, China). Hematoxylin (DH0001) used for immunohistochemical staining was purchased from LEAGENE (Beijing, China). BCA protein assay kit (P0011) was obtained from Beyotime (Shanghai, China). Elastic Van Gieson staining kit (BA-4083B) was purchased from BASO (Zhuhai, China).

**Enzyme-linked Immunosorbent Assay (ELISA)**

A human COMP ELISA Kit (DCMP0) and a mouse COMP ELISA kit (SEB197Mu) were used to measure the plasma COMP level of patients and mice, respectively. ELISA was performed according to the manufacturer’s protocols.

**Echocardiography**

Echocardiograms were performed using a Visual Sonics Vevo 2200 imaging system (VisualSonics, Toronto, Canada). Wild type male mice, *Fbn1^C1041G/+^* male mice and *Fbn1 ^C1041G/+^ COMP^SM-Tg^* male mice at 16 and 30 weeks were anaesthetized with 2% isoflurane to maintain heart rate at 400-500 beats/min. The diameters of the aortic root were assessed from B-mode of the parasternal long-axis view and measured between the inner edge to inner edge of the vessel at the end diastole from three separate heart beats.

**Western Blotting**

The thoracic aorta of 7-day 0.3% BAPN treated mice and 30-week-old *Fbn1^C1041G/+^* mice was frozen, crushed and lysed in RIPA buffer supplemented with PMSF. Protein concentrations were evaluated using a BCA protein assay kit (P0011). Western blotting was performed as previously described^1^. Quantitative data were obtained by gray value measurement via Image J.

**Immunohistochemical Staining**

Classic immunohistochemical staining for COMP was performed with a staining kit (PV-6001) according to the manufacturer’s protocol. Briefly, the frozen sections of mouse and human thoracic aorta were incubated with 3% v/v H_2_O_2_ and then blocked with 3% BSA. The sections were incubated with primary antibody against COMP (1:300, ab42225) at 4°C for 12 hours, followed by secondary antibody incubation before being stained with a DAB kit (ZLI-9018). Nuclei were subsequently stained with hematoxylin (DH0001).

For immunohistochemical staining of human COMP, 3 non-TAD samples and 3 TAD samples were used as previously described^2^**.** Briefly, segments of full-thickness and dissected ascending aortas were freshly and immediately harvested during surgery. The full-thickness ascending aortas segments from 3 non-TAD samples were served as controls. Two of them underwent dilated cardiomyopathy, and one had arrhythmia cardiomyopathy. The detailed information of all the patients is listed in **Table S4**.

**Immunofluorescence Staining**

The expression level of MMP2, MMP9, CD45 and COMP was assessed by immunofluorescence staining. Briefly, the frozen sections of mouse were incubated with 3% BSA for 1 hour and antibody against MMP2 (1:100, 10373-2-AP), MMP9 (1:100, 10375-2-AP) or CD45 (1:100, 103107) at 4℃ for 12 hours. The sections of thoracic aorta from MFS patients or controls were incubated with antibody against COMP (ab42225, Abcam) at 4℃ for 12 hours. Then, the sections incubated with antibody against MMP2, MMP9 and COMP were incubated with Alexa Fluor 633-conjugated goat anti-rabbit secondary antibody at room temperature for 1 hour. Nuclei were incubated with DAPI at room temperature for 10 minutes.

For immunofluorescence staining of human COMP, 3 non-TAD samples^2^ and 3 MFS samples were used as previously described^3^**.** The detailed information of all the patients is listed in **Table S7**.

**Elastic Van Gieson Staining and Degradation Score Evaluation**

Aortic elastin was stained by elastic Van Gieson staining (BA-4083B) according to the manufacturer’s protocol. Elastin degradation was graded as described in previous reports^2^. Specifically, level 1 means < 25% degradation; level 2 means 25% to 50% degradation; level 3 means 50% to 75% degradation; and level 4 means > 75% degradation.

**Reference**

1. Kessler T, Zhang L, Liu Z et al. ADAMTS-7 Inhibits Re-endothelialization of Injured Arteries and Promotes Vascular Remodeling Through Cleavage of Thrombospondin-1. *Circulation* 2015;131:1191-1201.
2. Yang X, Xu C, Yao F et al. Targeting endothelial tight junctions to predict and protect thoracic aortic aneurysm and dissection. *Eur Heart J* 2023;44:1248-1261.
3. Huang J, Liu H, Liu Z et al. Inhibition of aortic CX3CR1+ macrophages mitigates thoracic aortic aneurysm progression in Marfan syndrome in mice. *J Clin Invest* 2025;135.


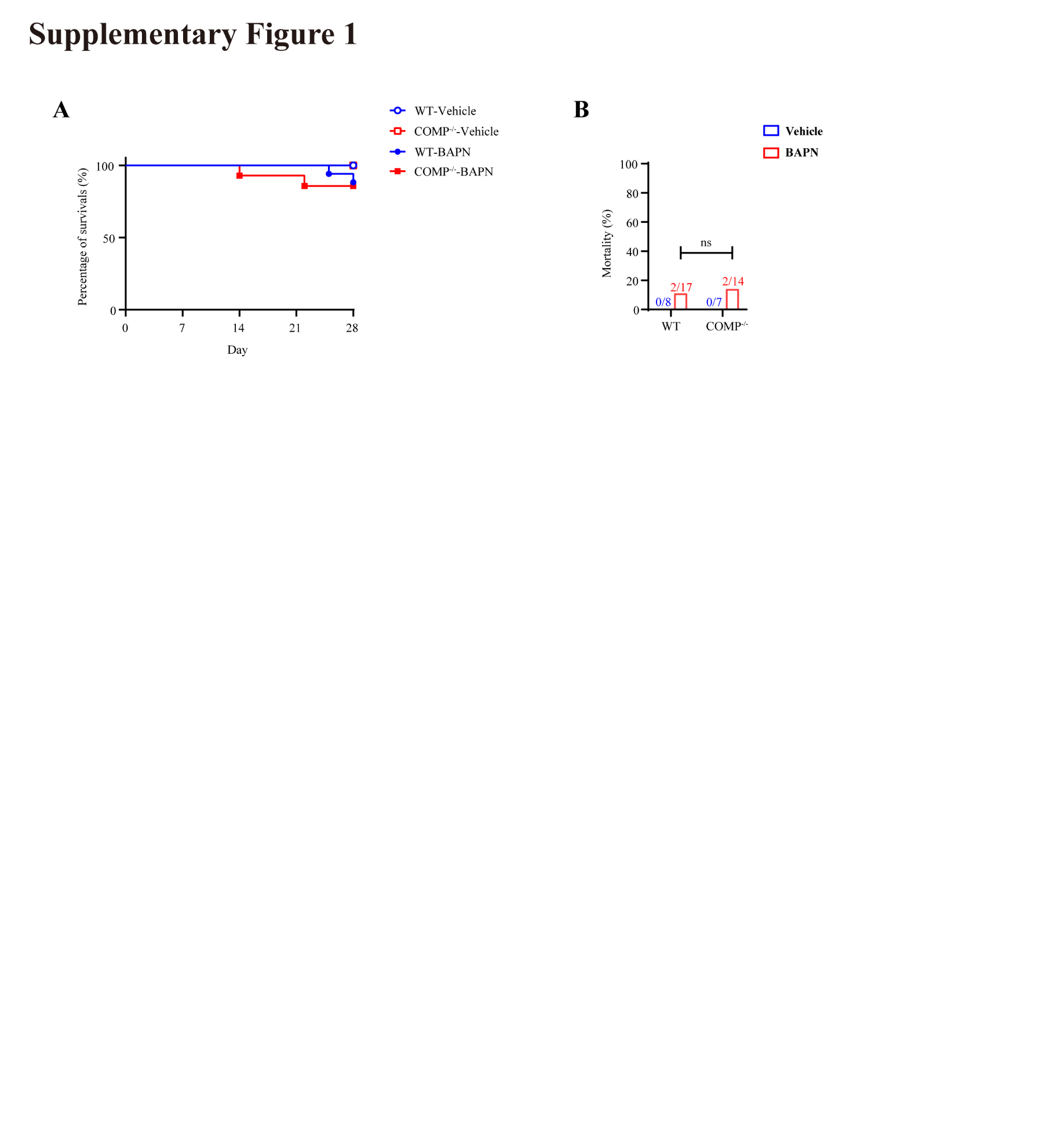


**Supplemental Figure 1.** (A-B) Survival curve and analysis of 28-day 0.3% BAPN-treated (BAPN) or control (Vehicle) WT or *COMP^-/-^* male mice. NS, no significance by Fisher’s exact test.


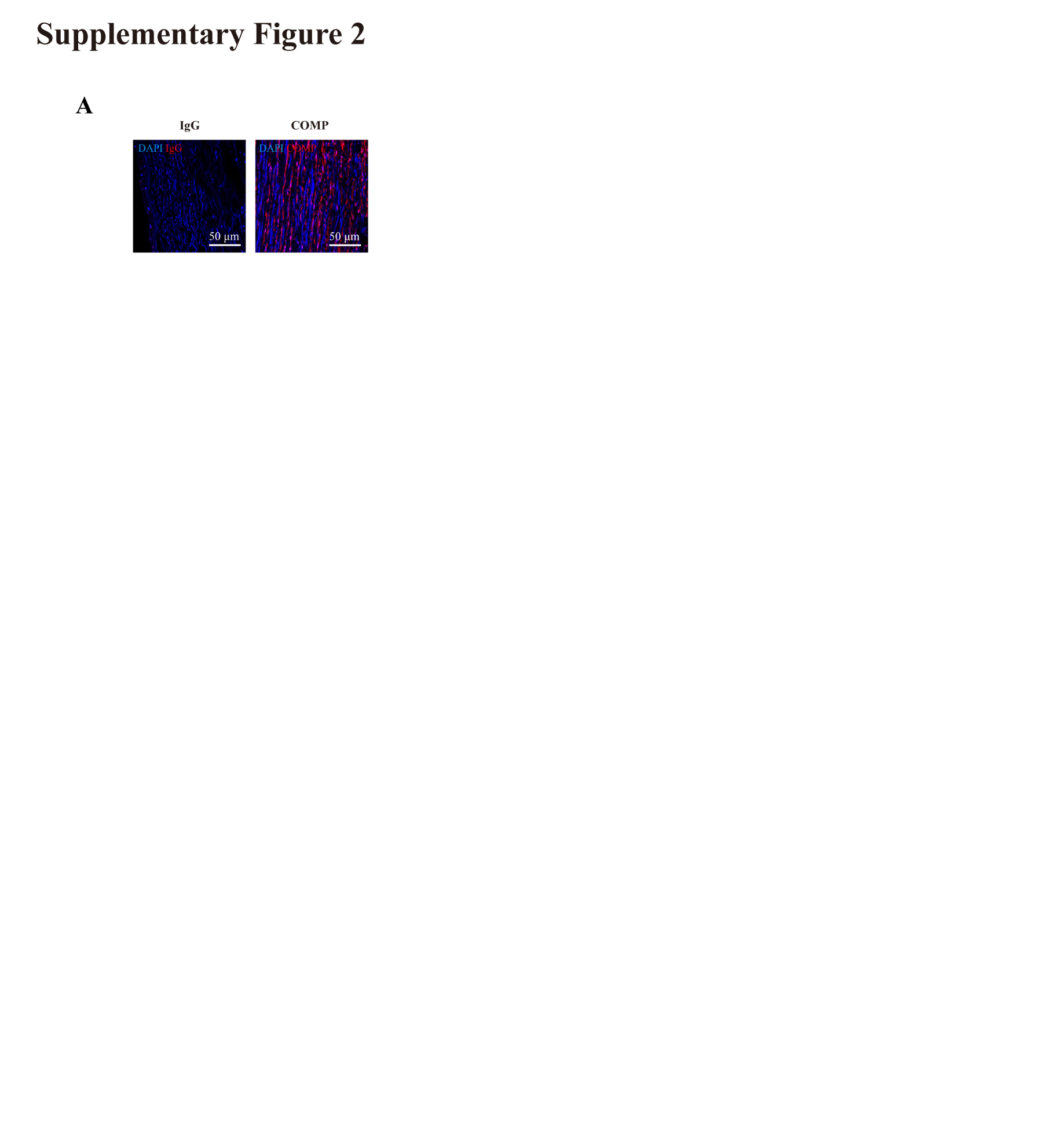


**Supplemental Figure 2.** (A) Representative immunofluorescence staining of COMP in ascending aortas from controls (Scale bar = 50 μm as shown in the image).


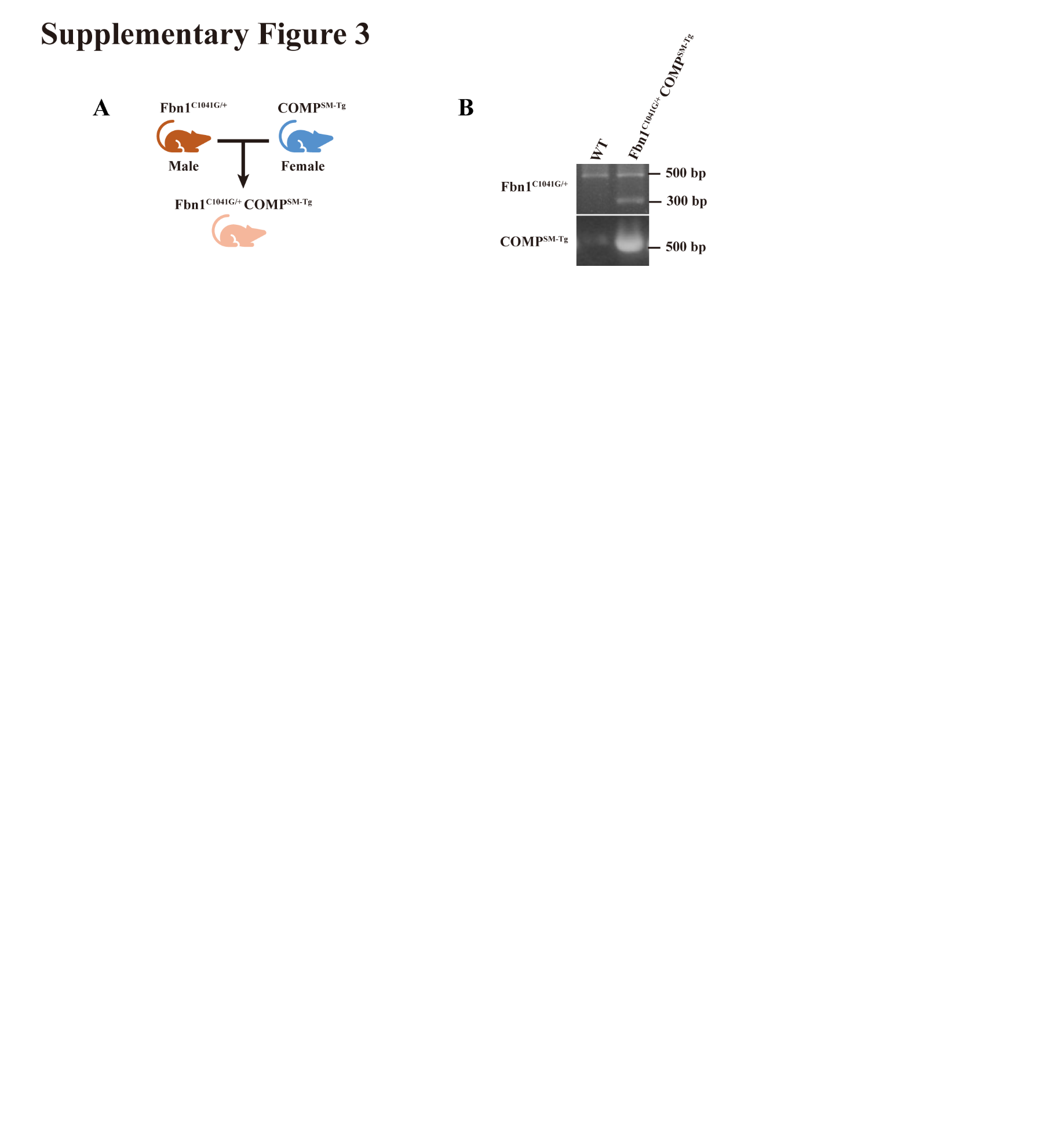


**Supplemental Figure 3.** (A) Schematics of crossing *Fbn1^C1041G/+^* mice with *COMP^SM-Tg^* mice. (B) Representative genotyping of *Fbn1^C1041G/+^ COMP^SM-Tg^* mice.

**Supplemental Table 1. ROC analyses of plasma COMP in TAD patient compared with healthy controls**

|  | **Total TAD** | **Type A** | **Type B** |
| --- | --- | --- | --- |
| Area | 0.742 | 0.726 | 0.756 |
| 95% CI | 0.698-0.785 | 0.670-0.782 | 0.705-0.807 |
| *P* value | <0.001* | <0.001* | <0.001* |

ROC, receiver operating characteristic; CI, confidence interval; COMP, cartilage oligomeric matrix protein; TAD, thoracic aortic dissection. **P*<0.05 by receiver operating characteristic analyses.

**Supplemental Table 2. Clinical and biochemical characteristics of Type A and Type B TAD patients included in the study respectively**

|  | **TAD** | | **Control** |
| --- | --- | --- | --- |
|  | **Type A** | **Type B** |  |
| N | 169 | 193 | 136 |
| Sex (Male) | 137 (81.1%) | 160 (82.9%) | 102 (75.0%) |
| Age (year) | 51.82±0.78*** | 54.40±0.79** | 58.05±1.00 |
| Hypertension | 83 (49.1%)^###^ | 91 (47.2%)^###^ | 34 (25.0%) |
| Smoking | N=169  48 (28.4%) | N=193  58 (30.0%) | N=99  29 (29.3%) |
| Drinking | N=169  26 (15.4%) | N=193  32 (16.6%) | N=99  19 (19.2%) |
| Blood Glucose  (mmol/L) | N=132  7.68±0.27** | N=170  7.32±0.17*** | N=127  5.84±0.11 |
| COMP  (ng/mL) | 98.84±7.33*** | 90.64±6.59*** | 155.50±12.95 |

TAD, thoracic aortic dissection; COMP, cartilage oligomeric matrix protein. ***P*<0.01, ****P*<0.001 by Kruskal-Wallis test with Dunn’s multiple comparisons test compared to Control. ^###^*P*<0.001 by χ^2^ test compared to Control. Data are shown as the mean ± SEM or count (%).

**Supplemental Table 3. OR analysis of plasma COMP in total TAD patients with different subgroups.**

| **Total TAD** | | **OR** | **95% CI** | ***P* value** |
| --- | --- | --- | --- | --- |
| Total | | 0.995 | 0.993-0.997 | <0.001* |
| Gender | Male | 0.995 | 0.992-0.997 | <0.001* |
|  | Female | 0.997 | 0.993-1.000 | 0.076 |
| Age | < 60 | 0.995 | 0.992-0.998 | <0.001* |
|  | ≥ 60 | 0.996 | 0.993-0.999 | 0.010* |
| Hypertension | - | 0.993 | 0.989-0.996 | <0.001* |
|  | + | 0.999 | 0.995-1.003 | 0.71 |
| Smoking | - | 0.997 | 0.995-0.999 | 0.002* |
|  | + | 0.996 | 0.992-1.000 | 0.079 |
| Drinking | - | 0.997 | 0.995-0.999 | 0.002* |
|  | + | 0.992 | 0.984-1.001 | 0.075 |
| Blood Glucose | < 7 | 0.997 | 0.989-0.997 | <0.001* |
|  | ≥ 7 | 0.996 | 0.993-1.000 | 0.026* |

OR, odds ratio; CI, confidence interval; COMP, cartilage oligomeric matrix protein; TAD, thoracic aortic dissection. **P*<0.05 by univariable logistic regression analysis.

**Supplemental Table 4. Patient information for vascular immunohistochemical staining.**

|  | **Control 1** | **Control 2** | **Control 3** | **TAD 1** | **TAD 2** | **TAD 3** |
| --- | --- | --- | --- | --- | --- | --- |
| Age | 54 | 62 | 28 | 41 | 42 | 53 |
| Gender | Female | Male | Male | Male | Male | Female |
| Diagnosis | Dilated cardiomyopathy | Dilated cardiomyopathy | Arrhythmia cardiomyopathy | Type A  TAD | Type A  TAD | Type A  TAD |
| Diabetes mellitus | No | Yes | No | No | No | No |
| Hypertension | No | No | No | Yes | No | Yes |
| Smoking | No | No | Yes | No | No | No |
| Alcohol | No | No | No | No | Yes | No |

TAD, thoracic aortic dissection.

**Supplemental Table 5. Weights and aortic diameters of 7-week-old WT mice or *COMP^-/-^* mice after 28-day vehicle or 0.3% BAPN treatment.**

|  | **WT-vehicle** | **WT-BAPN** | ***COMP^-/-^*-vehicle** | ***COMP^-/-^*-BAPN** |
| --- | --- | --- | --- | --- |
| n | 8 | 17 | 7 | 14 |
| Weight (g) | 14.62±0.67 | 14.74±0.52 | 14.64±0.50 | 14.96±0.60 |
| Maximum diameters of ascending aorta (mm) | 1.03±0.04 | 1.13±0.05 | 1.02±0.04 | 1.51±0.09*** |
| Maximum diameters of aortic arch (mm) | 1.05±0.04 | 1.21±0.09 | 0.97±0.03 | 1.85±0.13*** |
| Maximum diameters of descending aorta (mm) | 0.97±0.02 | 1.10±0.08 | 0.89±0.02 | 1.51±0.14** |

WT, wild type; COMP, cartilage oligomeric matrix protein. ***P*<0.01, ****P*<0.001 compared to WT-BAPN by two-way ANOVA with Sidak’s multiple comparisons test. Data are shown as the mean ± SEM.

**Supplemental Table 6. Weights and SBP of 30-week-old WT mice and *Fbn1^C1041G/+^* mice.**

|  | **WT** | ***Fbn1^C1041G/+^*** | ***P* value** |
| --- | --- | --- | --- |
| n | 6 | 6 |  |
| Weight (g) | 28.83±3.31 | 31.83±2.04 | 0.088 |
| SBP (mmHg) | 118.60±15.74 | 117.30±15.11 | 0.82 |

WT, wild type; COMP, cartilage oligomeric matrix protein; SBP, systolic blood pressure. Groups compared using *t* test. Data are shown as the mean ± SEM.

**Supplemental Table 7. Patient information for vascular immunofluorescence staining.**

|  | **Control 1** | **Control 2** | **Control 3** | **MFS 1** | **MFS 2** | **MFS 3** |
| --- | --- | --- | --- | --- | --- | --- |
| Age | 54 | 62 | 28 | 15 | 26 | 33 |
| Gender | Female | Male | Male | Male | Male | Female |
| Diagnosis | Dilated cardiomyopathy | Dilated cardiomyopathy | Arrhythmia cardiomyopathy | Aortic aneurysm A3C,  Aortic root aneurysm | Aortic root aneurysm,  Aortic regurgitation | Thoracoabdominal aortic aneurysm  (Crawford Type III) |
| Diabetes mellitus | No | Yes | No | No | No | No |
| Hypertension | No | No | No | No | No | No |
| Smoking | No | No | Yes | No | No | No |
| Alcohol | No | No | No | No | No | No |

MFS, Marfan syndrome.

**Supplemental Table 8. Weights and SBP of 30-week-old *Fbn1^C1041G/+^* mice and *Fbn1^C1041G/+^* *COMP^SM-Tg^* mice.**

|  | ***Fbn1^C1041G/+^*** | ***Fbn1^C1041G/+^ COMP^SM-Tg^*** | ***P* value** |
| --- | --- | --- | --- |
| n | 6 | 9 |  |
| Weight (g) | 31.83±2.04 | 29.78±5.21 | 0.38 |
| SBP (mmHg) | 117.30±15.11 | 119.90±15.69 | 0.64 |

COMP, cartilage oligomeric matrix protein; SBP, systolic blood pressure. Groups compared using *t* test. Data are shown as the mean ± SEM.
